# P-2328. Influenza and RSV Surveillance in the US Veterans Health Administration (VHA): 2023-2024 Season

**DOI:** 10.1093/ofid/ofae631.2480

**Published:** 2025-01-29

**Authors:** Cynthia A Lucero-Obusan, Joyce S Chung, Patricia Schirmer, Connor W Edson, Gina Oda, Mark Holodniy

**Affiliations:** U.S. Department of Veteran Affairs, Public Health National Program Office, Palo Alto, California; VHA Public Health National Program Office, Palo Alto, California; Department of Veterans Affairs, Palo Alto, California; Department of Veterans Affairs, Palo Alto, California; Department of Veterans Affairs, Palo Alto, California; Department of Veterans Affairs, Palo Alto, California

## Abstract

**Background:**

Influenza and Respiratory Syncytial Virus (RSV) cause seasonal epidemics with substantial morbidity and mortality. Veterans Health Administration’s (VHA) large elderly population is at higher risk for complications, including hospitalization and death. Herein we summarize VHA’s annual surveillance data for influenza and RSV activity and vaccinations.

**Figure:**
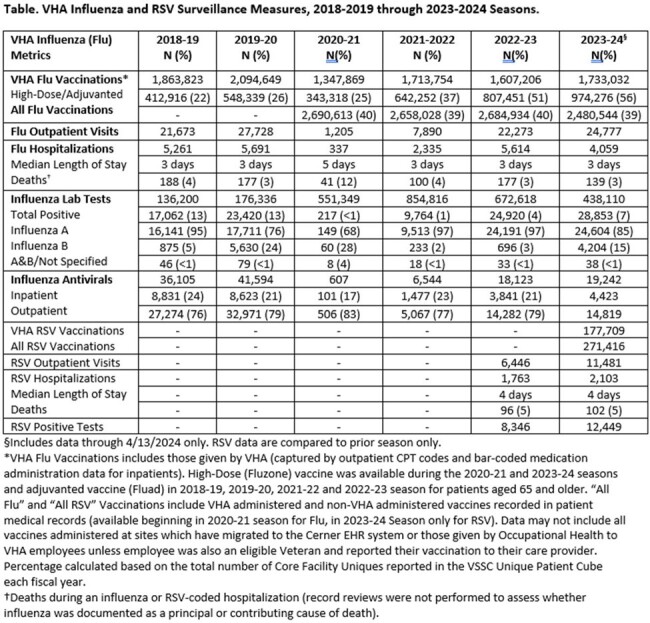

**Methods:**

Influenza-like-illness (ILI) as well as influenza and RSV-coded outpatient visits and hospitalizations, laboratory testing, and antivirals were obtained from VHA data sources (10/1/2023-4/13/2024) and compared to prior years and CDC data. VHA and community vaccinations were captured starting August 1^st^ each year, with rates calculated based on VHA users in each fiscal year. Influenza RT-PCR positive respiratory samples underwent whole genome sequencing (WGS) to analyze clade and antiviral resistance.

**Figure:**
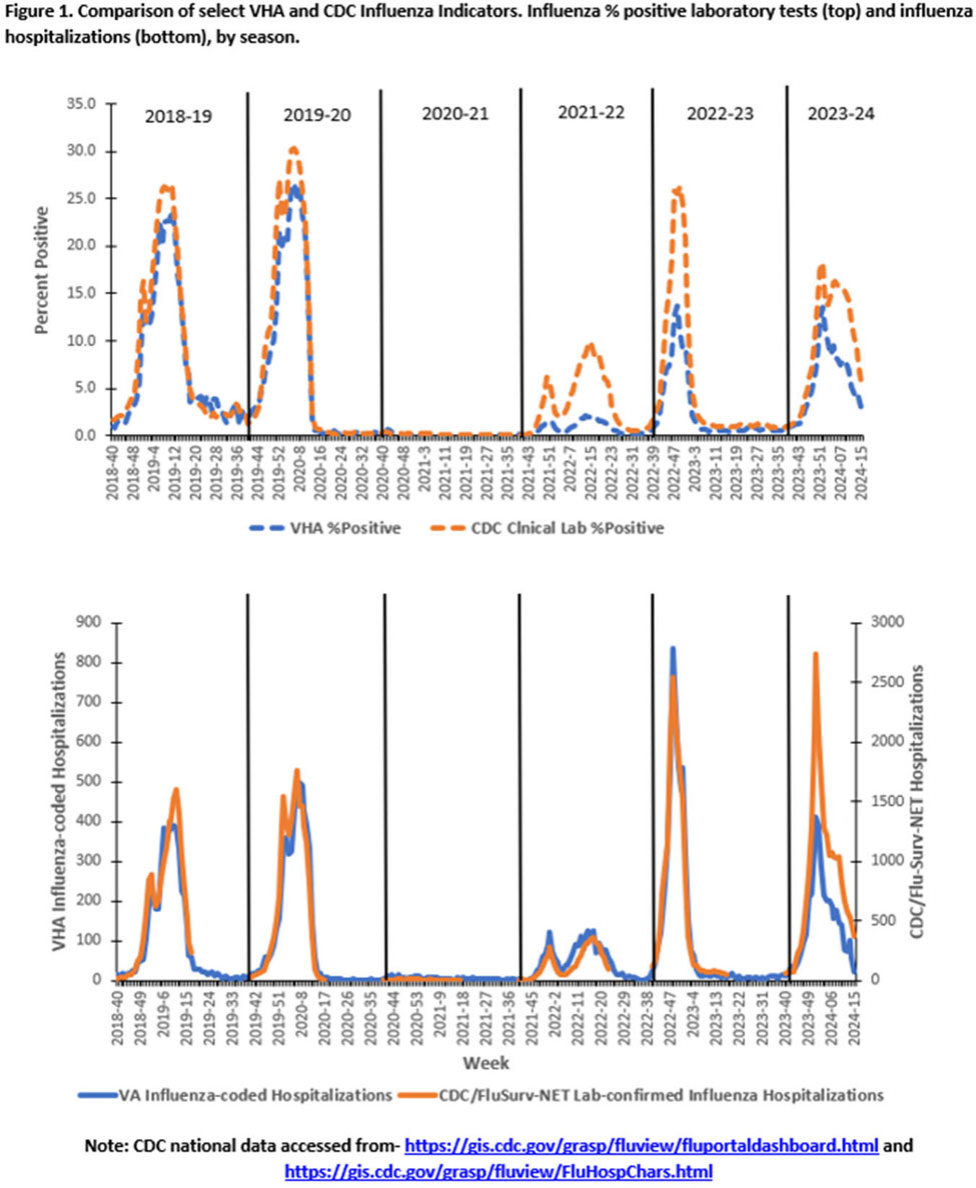

**Results:**

Influenza vaccinations (including high dose) were increased compared to 2022-2023 (Table). Weekly ILI ranged from 0.6%-1.6% in primary/urgent care settings. Activity was highest for 2023 Week 52, matching national influenza trends and increased compared to last season (Fig.1). There were 28,853 influenza positives among 438,110 tests performed (6.6%). Median length of stay was 3 days in 4,059 hospitalizations. Among 139 deaths, 129 (92.8%) had Influenza A and 10 (7.2%) Influenza B (Table, Fig.2). 310 samples (in 5 states) were sequenced with no drug resistance mutations identified (237 A/H1; 30 A/H3; 43 B/Victoria), representing 4 subclades, which aligned with CDC reporting (Fig.3). Over 270,000 Veterans received an RSV vaccine in 2023-2024 (over 177,000 administered by VHA). RSV visits, hospitalizations and positive tests were increased compared to 2022-2023.

**Figure:**
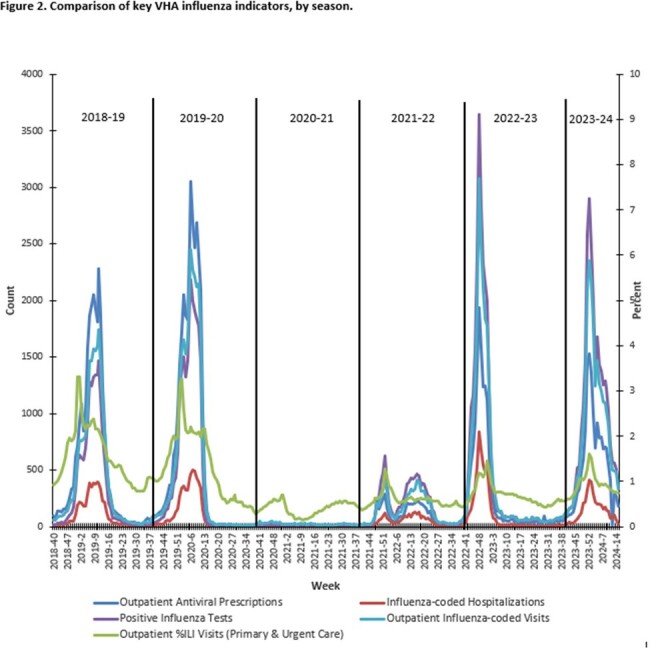

**Conclusion:**

For 2023-2024, influenza vaccinations (including high dose) increased compared to prior years, and newly licensed RSV vaccines were available in VHA. Influenza and RSV activity was increased compared to the prior season. VHA influenza activity and WGS analysis tracks closely with national CDC reporting. Given risks associated with influenza and RSV in the elderly population, VHA will continue efforts to improve vaccination rates.

**Disclosures:**

All Authors: No reported disclosures

